# Synthesise and Characterization of Cordierite and Wollastonite Glass—Ceramics Derived from Industrial Wastes and Natural Raw Materials

**DOI:** 10.3390/ma15103534

**Published:** 2022-05-14

**Authors:** Gamal A. Khater, Amany A. El-Kheshen, Mohammad M. Farag

**Affiliations:** Glass Research Department, National Research Centre, Cairo 12622, Egypt; aelkheshen1@yahoo.com (A.A.E.-K.); mmfaragnrc@gmail.com (M.M.F.)

**Keywords:** crystallization, glass–ceramics, diopside, β-wollastonite, parawollastonite, anorthite, by-pass cement

## Abstract

Industrial waste is one of the primary sources that harm the environment, and this topic has occupied many scientists on how to take advantage of these wastes or dispose of them and create a clean environment. By-pass cement dust is considered one of the most dangerous industrial wastes due to its fine granular size and its volatilization in the air, which causes severe environmental damage to human and animal health, and this is the reason for choosing the current research point. In this article, eight samples of glass–ceramics were prepared using by-pass cement dust and natural raw materials known as silica sand, magnesite, and kaolin. Then melted by using an electric furnace which was adjusted at a range of temperatures from 1550 to 1600 °C for 2 to 3 h; the samples were cast and were subjected to heat treatment at 1000 °C for 2 h based on the DTA results in order to produce crystalline materials. Various techniques were used to study the synthesized glass–ceramic samples, including differential thermal analysis (DTA), X-ray diffraction (XRD), scanning electron microscope (SEM), and thermal expansion coefficient (CTE). X-ray analysis showed that the phases formed through investigated glass–ceramic samples consisted mainly of β- wollastonite, parawollastonite, diopside, anorthite, and cordierite. It was noticed that β- the wollastonite phase was formed first and then turned into parawollastonite, and also, the anorthite mineral was formed at low temperatures before the formation of the diopside mineral. SEM showed that the formed microstructure turned from a coarse grain texture to a fine-grained texture, by increasing the percentage of cordierite. It also showed that the increase in time at the endothermic temperature significantly affected the crystalline texture by giving a fine-grained crystalline texture. The linear thermal expansion measurements technique used for the studied glass–ceramic samples gives thermal expansion coefficients ranging from 6.2161 × 10^−6^ to 2.6181 × 10^−6^ C^−1^ (in the range of 20–700 °C), and it decreased by increasing cordierite percent.

## 1. Introduction

Since the dawn of time, the industry has been considered one of the most important pillars that depend on the consumption of materials, the production of all that humanity needs for the well-being of life, and also is assumed as one of the most crucial reasons for the existence of waste materials.

From the financial and ecological points of view, the recycling of manufacturing wastes is well thought-out to be a significant issue. The increase of industrial waste materials, due to the rapid increase in populations and growth in the prosperity of humanity, has become a major social and environmental problem. Management of all industrial waste has become the most significant environmental issue in numerous expanding countries. Primary sources of solid waste are human liveliness, such as building and technology innovation. These wastes cause many complex problems concerning storage space, transportation, and environmental or atmosphere contamination. Utilizing manufacturing waste to produce beneficial resources via green chemical methods will be an essential step to having a hygienic and safe environment [1,2].

Cement is one of the most remarkable binding materials. It is manufactured in massive amounts all over the world. The cement industry is considered one industry that generates an enormous amount of solid waste. These wastes should be managed to guarantee a clean and harmless environment [3].

In Egypt, several cement factories create significant amounts of cement kiln dust (CKD), the by-product formed in cement kilns and associated procedures. It has a destructive outcome on all living organisms [4]. The chemical composition of CKD mainly depends on the composition of raw materials, speed of the gas in the kiln, and type of procedure. Usually, CKD contains calcined materials, un-reacted raw feed, clinker dust, ash, alkali sulfates, halides, and other volatile materials [5,6,7,8].

Efforts have been made by several researchers to recycle that harmful CKD waste through different valuable applications such as removal of heavy metals [9], treating contaminated soil [10], ceramic production [11], glass–ceramic fabrication [12,13,14], and so on. 

The chemical composition of by-pass cement dust qualifies it for the glass–ceramic and glass industries [15]. As the glass–ceramics industry has various applications, such as in the microelectronics industry and construction. Utilizing cement dust to produce glass–ceramic materials is highly considered a technological, scientific, and cost-effective concept.

Glass–ceramic is one of the majority multipurpose and valuable materials needed in diverse industrial applications. Glass-ceramic has varieties of properties according to its composition, such as hardness, insulation, thermal shock resistance, low thermal expansion coefficient, toughness, and optical properties [16]. It is usually prepared by scheming glass crystallization. It also has fine-grained microstructure, low or no porosity, and lots of different properties that may be synchronized by changes in sample composition and the regime of heat treatment. 

Wollastonite (CaSiO_3_) has been conventionally applied as a raw material for tiles, paint, paper industries [17], insulators [18], ceramics [19], and so on. It has good mechanical, thermal, and optical properties such as slight shrinkage, high strength, whiteness in color, and bending properties. Nowadays, the increasing demands for wollastonite are followed by the proportional increases in its fabrication worldwide [20]. Natural wollastonite is limited by its deposits condition so that it can be synthesized artificially [17]. Synthesized wollastonite glass–ceramics [21] are used for various purposes and must be studied. 

Cordierite (2MgO–2Al_2_O_3_–5SiO_2_) is one of the most motivating phases of glass–ceramics; and has a broad collection of applications in many industrial areas [22]. It possesses features such as low thermal expansion, high chemical durability, high thermal resistance, low dielectric constant, and good mechanical properties, which give scientists the facility to use this compound to produce traditional or advanced products [23,24]. Cordierites have lower production costs and superior electrical properties, they can be used as substrate more than alumina in electronic industries and manufacturing multilayer circuit boards and thermal insulators.

This research aims to take advantage of cement dust as one of the industrial wastes produced in huge quantities from cement factories with some raw materials available in the earth’s crust to produce high-performance glass–ceramic materials that can be used for construction applications. The purpose of the research is to take advantage of by-pass cement dust, one of the solid industrial wastes which harm the environment, in obtaining high-performance glass–ceramic materials based on the wollastonite–cordierite system that can be used for various industrial and construction purposes by using many techniques such as differential thermal analysis (DTA), X-ray diffraction (XRD), scan electron microscope (SEM: JEOL, XL30, Philips, Amsterdam, The Netherlands)), and thermal expansion coefficient (CTE).

## 2. Experimental Techniques

### 2.1. Batch Calculation and Glass Preparation

Eight glass compositions were designed based on cordierite (Mg_2_Al_4_Si_5_O_18_) and wollastonite (CaSiO_3_) systems selected for the current project. The cordierite content in these compositions varies from 10 to 80 at ten wt% intervals, and consequently, the wollastonite content ranges from 90% to 20%. These studied samples were G10, G20, G30, G40, G50, G60, G70, and G80. These numbers denote the wt% of the cordierite and the rest being the wollastonite components. For example, G40 means that this glass composition contains 40wt% cordierite component and the rest, 60wt% wollastonite component. The batches related to these compositions were designed by calculating the appropriate proportions of solid wastes represented in by-pass cement dust, magnesite, kaolin, and silica sand. Table 1 shows the chemical compositions of the raw materials used. Table 2 shows glass compositions in the oxide percentages and raw materials used. These batches were ground in a ball mill for about 45 min, then they were melted in platinum crucibles in an electrically heated globar furnace at temperatures ranging from 1550 °C to 1600 for 2–3 h, according to the batch composition. It was noted that glasses rich in cordierite were highly viscous and needed higher melting temperatures. The melting temperature increased with an increase in the percentage of cordierite contents (that is, the degree of viscosity increases from G10 to G80).

After melting and refining, the resulting bubble-free melts were cast onto a hot steel marver into steel molds in the form of buttons (about 20 mm and 9 mm thickness) as rods of 50 × 10 ×10 mm. The hot glass samples were transferred to preheated electric muffle furnace to avoid thermal shock.

### 2.2. Differential Thermal Analysis (DTA)

The differential thermal analyses of the studied glasses were achieved via a Labsys DSC-TG 1600 °C Setaram (France). About 60 mg of the pulverized glass of grain size less than 0.60 mm and more significant than 0.2 mm was used. Al_2_O_3_ powder was the reference material. A heating rate of 10 °C/min and a sensitivity setting of 8 μv/cm were maintained for all the DTA runs. 

### 2.3. Heat Treatment

**Figure 1:**
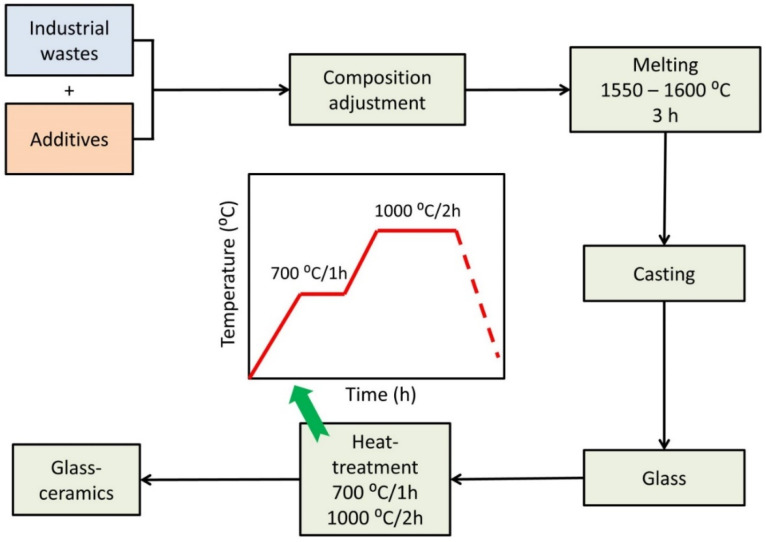
Schematic presentation for the production of glass and glass–ceramic materials from industrial wastes.

The glass samples were heat-treated from room temperature to the required temperature for a specific soaking time in a muffle furnace, then the furnace was switched off, and the samples were permitted to cool inside it to room temperature. Detection of the crystallization temperatures was guided by the DTA results. Soaking times were measured after the furnace reached the desired temperature. Figure 1 shows Schematic presentation for the production of glass and glass–ceramic materials from industrial wastes.

### 2.4. X-ray Diffraction (XRD)

The formed phases were recognized via the X-ray diffraction technique. The instrument used was Bruker AXS diffractometer (CD8—ADVANCE) with Cu–Ka radiation operating at 40 Kv and ten mA. The diffraction information was listed as 2θ values between 4° and 70°, and the scanning rate was 10°/min.

### 2.5. Scanning Electron Microscope (SEM)

The microstructure of the synthesized glass–ceramic samples was investigated by using a scanning electron microscope (SEM: JEOL, XL30, Philips, Amsterdam, The Netherlands), that operated at an acceleration voltage of 20 kV. The freshly fractured sample was coated with a layer of gold on the broken surface (to minimize any charging effect) to observe the internal microstructure.

### 2.6. Thermal Expansion Measurement

In this study, the mechanical dilatometry technique for thermal expansion measurement is applied. In this technique, a sample is heated in an oven, and the specimen ends; displacements are transmitted to a sensor utilizing pushrods. This test is usually functionalized to materials with CTE above 5 × 10^−6^/K (2.8 × 10^−6^/°F) over the temperature range of −180 to 900 °C (−290 to 1650 °F). Pushrods are of high-purity alumina type. Alumina systems can extend the temperature range up to 1600 °C (2900 °F). The instrument used was NETZSCH DIL 402 PC (made in Japan).

## 3. Results and Discussion

### 3.1. Differential Thermal Analysis (DTA)

**Figure 2:**
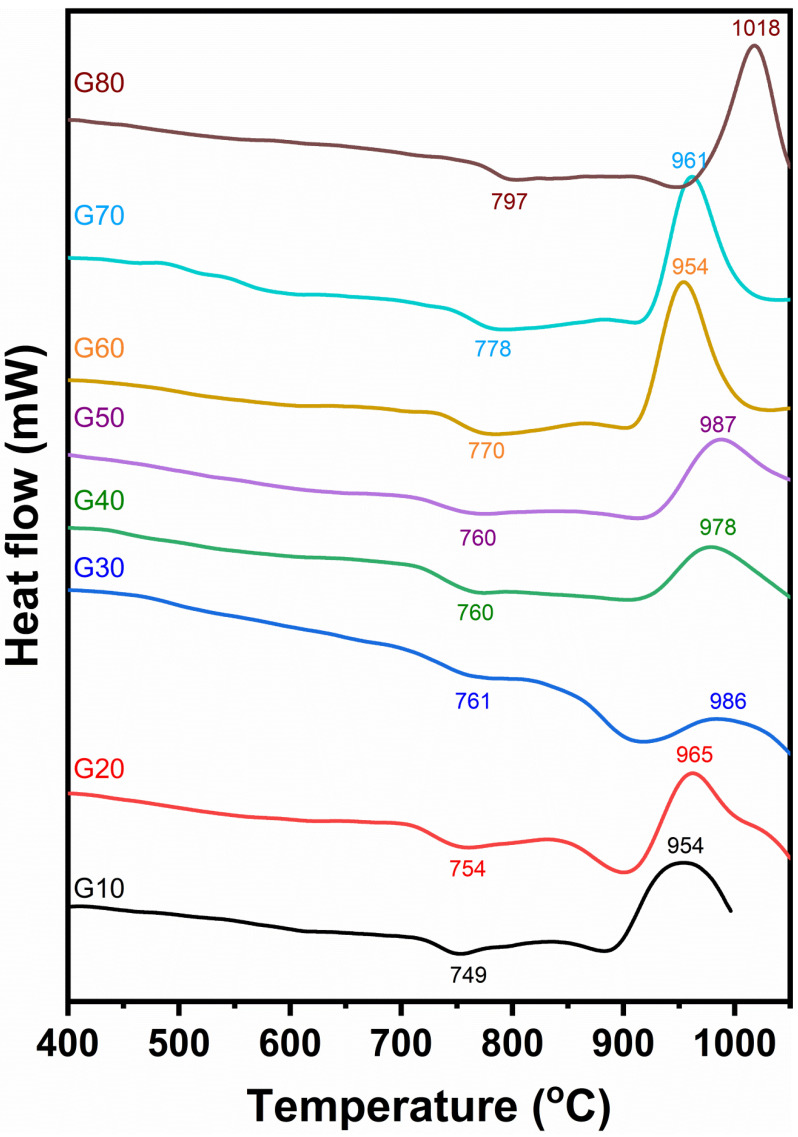
Differential thermal analysis of the investigated glasses.

DTA of the studied glasses is explained in Figure 2. The curves resulting from the investigated glasses showed endothermic peaks in the temperatures ranging from 749 to 797 °C. The differential thermal analysis curves show that the endothermic peaks of the investigated glasses are wholly affected by the cordierite contents in the glass. As the cordierite content increases, the endothermic peak temperatures shift towards higher values. The endothermic and exothermic peaks of the investigated glasses for each glass composition are shown in Table 3. The DTA of the glasses G10 to G50 (Figure 2) are, to some extent, similar. They are characterized by a broad exothermic peak with a temperature ranging from 954 to 987 °C. While the other glasses, G60 to G80, have characterized sharp exothermic peaks recorded at temperatures ranging from 954 to 1018 °C, respectively.

It should be said that the data recorded from DTA are considered to be the responsible guide for the heat treatment schedule of the investigated glasses.

The DTA curves show a slight dip in the range 749 to 797 °C most probably, as confirmed above, owing to the transition temperature of the glass (T_g_) or corresponding approximately, following Devekey and Majumdar [25], to the temperature range named as T_g_ and T_s._

These endothermic agree to initial crystallization (precrystallization), where the glass-creating oxides begin to arrange themselves at this period in beginning structure groups appropriate for the subsequent crystallization El-Shennawi [26]. This thermal absorption span will be, however, considered as the nucleation range. Exothermic peaks result from glass devitrification with a corresponding release of thermal energy.

### 3.2. X-ray Diffraction (XRD)

#### 3.2.1. XRD of the Investigated Samples after Treatment at Temperature 1000 °C for 2 h

**Figure 3:**
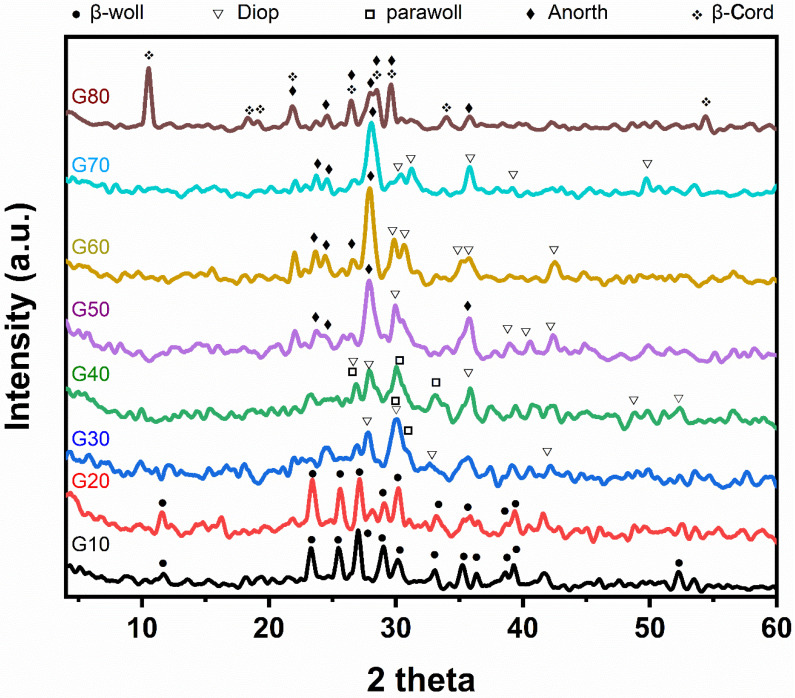
X-ray diffraction patterns of the investigated samples based on by-pass cement dust after heat-treatment at 1000 °C for 2 h.

X-ray diffraction analysis of the studied glass–ceramic samples G10–G80 after heat treatment at a temperature of 1000 °C for 2 h is shown in Figure 3. The main crystalline minerals developed are β-wollastonite (CaSiO_3_) (JSPDS No.27-1064), parawollastonite (CaSiO_3_) (JCPDS No.27-88), diopside (CaMgSi_2_O_6_) (JCPDS No.22-534), anorthite (CaAl_2_Si_2_O_8_) (JCPDS No.12-301) and cordierite (Mg_2_Al_4_Si_5_O_18_) (JCPDS No.13-294). Concerning samples G10 and G20, it is obvious that β- wollastonite (CaSiO_3_) is the only monomineralic phase formed, characterized by the following lines 3.296, 3.807, 3.489, and 3.076 Å. While for samples G30 and G40, the β-wollastonite transferred into parawollastonite, characterized by lines 2.97, 3.318, and 2.699 Å with the formation of the diopside phases, which is characterized by the following lines 2.972, 3.182, and 2.503 Å. Whereas in glass–ceramic samples Nos (G50–G70), the parawollastonite transforms into anorthite, characterized by the following lines 3.202, 4.036, 3.739, and 2.501 Å. In sample G80, the low-cordierite phase formed, which is characterized by the following lines, 8.412, 3.128, 3.366, and 3.015 Å with an anorthite phase. Samples G10 and G20 show the formation of β-wollastonite only; this is consistent with Table 2, where the percentage of wollastonite ranges from 90 to 80 % in samples G10 and G20, respectively. It also agrees with many publications, such as Shaker et al. [27] and Khater et al. [28,29], who explained that β-wollastonite is formed at low temperatures and is unstable and then transformed into parawollastonite. Samples G30 and G40 show the formation of parawollastonite and diopside. This shows that β-wollastonite (CaSiO_3_) is converted to parawollastonite, which agrees with Shaker et al. [27]. In samples G50, G60, and G70, the formation of diopside and anorthite is attributed to the aluminum elements present in cordierite with wollastonite, which help form the anorthite mineral while the magnesium and silica elements present in cordierite help in the formation of diopside. Sample G80 contains both minerals cordierite and anorthite, indicating the reach of a stage close to equilibrium phases.

#### 3.2.2. XRD of the G60 after Treatment at a Temperature

**Figure 4:**
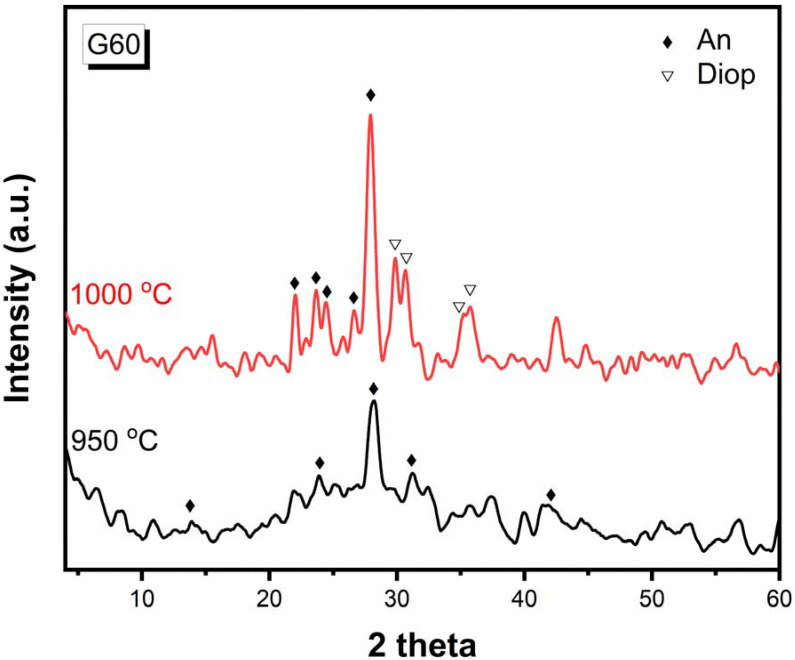
X-ray diffraction patterns of G60 after heat-treatment at 950 °C for 2 h and 1000 °C for 2 h.

Sample G60 is selected to study the effect of temperature on the crystalline phases formed by heat-treating. Sample G60 is heat-treated at a temperature of 950 °C for 2 h and also at 1000 °C for two hours (Figure 4). It is shown that after its heat treatment at 950 °C for 2 h, anorthite (CaAl_2_Si_2_O_8_) (JCPDS No.18-1202) is formed with the following lines 3.173, 4.013, 2.875, and 2.138Å. When the temperature is raised to 1000 °C, the mineral diopside is formed with anorthite, and this shows that the anorthite phase is formed before the diopside phase and at low temperatures. This agrees with Juan Qin et al. [30].

### 3.3. Scanning Electron Microscope (SEM)

**Figure 5:**
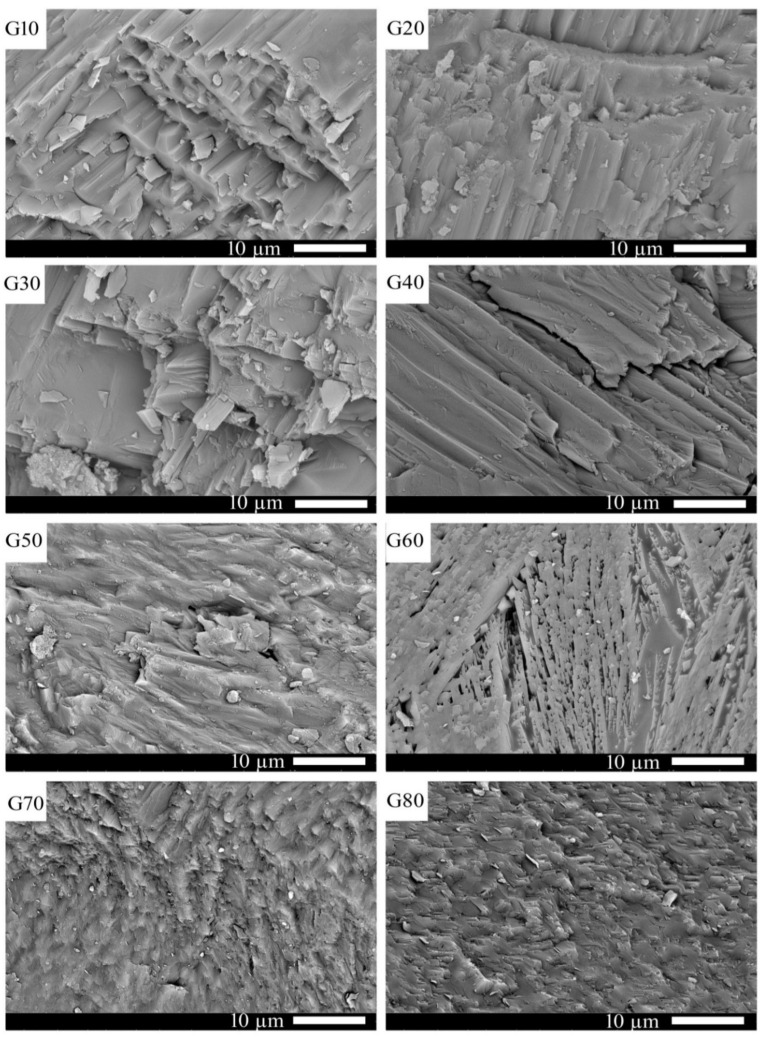
SEM micrographs of the investigated samples after heat-treatment at 1000 °C for 2 h.

Figure 5 shows SEM photographs of the glass–ceramics samples (G10 to G80) after heat treatment at 1000 °C for 2 h. It can be noticed from the figure that all samples are generally marked with well-crystallized oriented tubular crystals except sample G60, which is characterized by randomly oriented crystal bundles. Moreover, the crystal lengths are decreased from G10 to G80. These tubular crystals are identical to wollastonite crystals. Rounded crystals are noticed in the G80 sample, which is attributed to the formation of cordierite crystals, as confirmed by XRD analysis. The cordierite (Mg_2_Al_4_Si_5_O_18_) is nominally increased from G10 to G80 at the expense of the wollastonite (CaSiO_3_) phase in the cordierite–wollastonite system designed in this work. This increase in cordierite percentage increases the glass melt viscosity, as observed during the glass preparation process. This finding can be explained on the base of Al_2_O_3_ content, where it increased as the cordierite percentage increased in the glass composition. Al_2_O_3_ is capable of forming bonds with silica by forming bridging oxygens and generates Al–O–Si bonds causing an increase in glass viscosity [31]; hence, the glass crystallization affinity can be likely decreased, and the glassy phase is usually detected as a matrix for the formed crystals. In this study, the glassy phase can be observed in the crystal interstices, specifically in the G60 sample.

**Figure 6:**
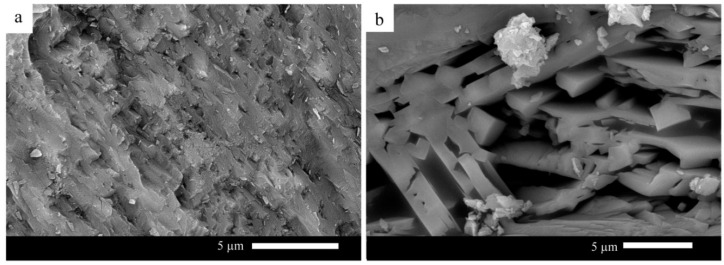
SEM micrographs of G60 heat-treated at (**a**) 700 °C 3 h and 1000 °C 1 h; (**b**) 700 °C 1 h and 1000 °C 1 h.

On the other hand, a selected glass sample was heat-treated at a temperature close to the nucleation temperature to study the effect of temperature heat treatment on the formed microstructure of the glass–ceramic. Sample G60 is selected because it is characterized by optimum properties measured in this study. Figure 6 presents G60 glass–ceramic heat-treated at 700 °C for 3 h and 1000 °C for 1 h. It can be observed from the figure that fine-grained microstructure is noticed. While the sample, after being heat-treated at 700 °C for one h and then 1000 °C, has a coarse-grained microstructure.

### 3.4. Theoretical Considerations of Thermal Expansion and Thermal Expansion Behavior of the Studied Samples

The coefficient of thermal expansion (CTE) is a property of a substance indicating the degree of material to be expanded by heating; substances expand at different altitudes. In the temperature ranges, the thermal expansion of objects is proportional to temperature alteration. Thermal expansion is an essential property for detecting the targeted application of materials when a structural part is heated and kept at a constant length [32].

Most solid substances expand by heating and contract after cooling. The change in length with temperature for a solid material can be expressed as:

(L_f_ − L_0_)/L_0_ = α_1_ (T_f_ − T_0_) (1)

ΔL/L_0_ = α_1_ΔT (2)

α_1_ = 1/L(dL/dT) (3)

where L_0_ and L_f_ stand for the original and final lengths sample with the temperature change from T_0_ to T_f_, respectively. The parameter α_1_ (CTE) has units of reciprocal temperature (K^–1^) such as µm/m K or 10^–6^/K.

Heating or cooling has a significant effect on the dimensions of a material, with a considerable volume change. Volume changes can be calculated from:

ΔV/V_0_ = α_v_ΔT (4)

where ΔV and V_0_ are the volume change and original volume of the sample, respectively, and α_v_ symbolizes the volume coefficient of thermal expansion. In many materials, the value of α_v_ is anisotropic; that is, it depends on the crystallographic orientation along which it is measured. For materials in which the thermal expansion is isotropic, α_v_ is approximately 3α_1_ [33].

Thermal expansion leads to a change in the space between particles of a substance, which affects the volume of the substance while negligibly varying its mass (the negligible amount comes from energy–mass equivalence).

**Figure 7:**
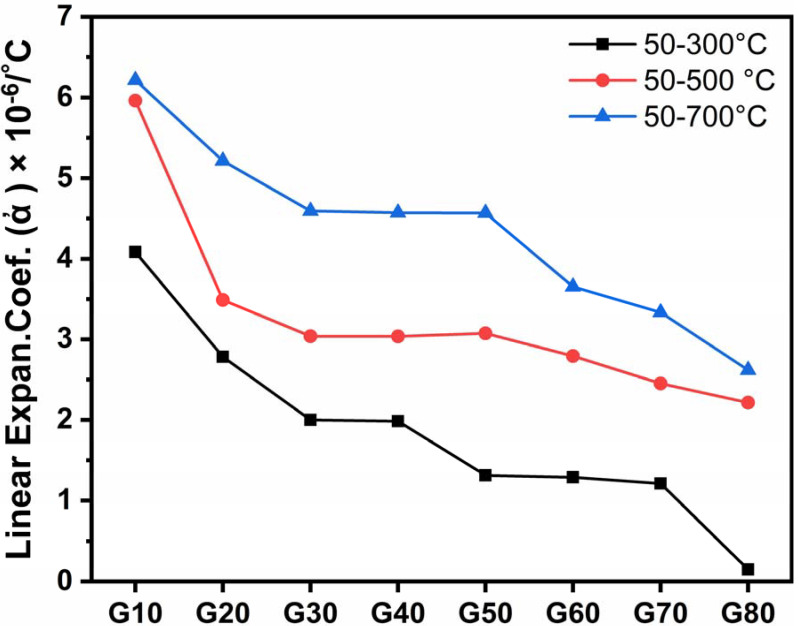
Thermal expansion coefficient of the studied glass–ceramics after heat-treatment at 1000 °C for 2 h.

Depending on the above considerations, the results of thermal expansion coefficients of the studied samples can be discussed as follows: 

From Figure 7 and Table 3, it is clear that all coefficients of thermal expansion (CTE) values decrease from G10 to G80 samples, i.e., by increasing the cordierite percent at the expense of wollastonite content.

Coefficient thermal expansion (CTE) values of the glass–ceramic depend on the nature and quantity of the developed crystalline phases and the residual glassy matrix [34,35]. A comprehensive range of thermal expansion coefficients is controlled by the crystal types and proportions of these phases, which are considered the main principle of producing glass–ceramics with restricted thermal expansion coefficients.

Low-expansion glass–ceramics usually contain crystalline phases as well as a vitreous phase. Ion exchange always takes place in the vitreous phase. Schairer and Bown [36] found a solid solution between wollastonite and diopside in the CaO–MgO–SiO_2_ system with the highest 22% diopside at eutectic. The produced Ca–Mg–silicate phase is a solid solution between diopside and wollastonite. Diopside constituent in this solid solution is about 49.6%. However, Omer et al. [37] mentioned that a wollastonite–diopside solid solution phase with about 66 % of diopside might be created. This Mg-rich wollastonite solid solution is eventually transformed by increasing time or raising the heat treatment temperature to wollastonite and diopside [38].

By increasing Al^3+^ ions (from G10 to G80), the formation of diopside, anorthite, and cordierite phases at the expense of wollastonite is noticed. Thus the values of the expansion coefficients (α) of glass–ceramic samples are decreased. Consequently, higher Tg temperatures could be expected. 

The thermal expansion property of the crystalline solids is relatively different from that of the parent glasses. The thermal expansion coefficient (α) of the glass–ceramics is a function of the thermal expansion coefficients, and elastic properties of all precipitated crystalline phases, residual glass, and the resulting microstructure. An extensive range of thermal expansion coefficients is covered by the different crystal types [39].

Wollastonite has an α-value of 94 × 10^−7^ °C^−1^ [40], but anorthite has α-values of 51–64 × 10^−7^ °C^−1^ [41]. 

Cordierite is well known for its very low thermal expansion coefficient of about 2.5 × 10^−6^/°C [42]. However, the CTE of cordierite may depend on the nature and amount of phases that coexist with it in the glass–ceramic sample [43]. The above α-values give a good explanation of the noticeable decrease in thermal expansion of the studied samples (Table 3 and Figure 7).

The thermal expansion coefficient (CTE) of the diopside is registered to be 83.0 × 10^−7^ °C^−1^ [44,45].

The co-existence of diopside, which has a high thermal expansion coefficient, with anorthite, which has a comparatively lower value of α, leads to a lowering of the thermal expansion coefficient of the final glass–ceramic product (G50–G70). 

In samples (G50–G80), it was observed that the presence of both CaO and Al_2_O_3_ containing crystalline phases leads to a decrease in the thermal expansion of the glass–ceramic samples. Al_2_O_3_ is mainly efficient in this concept. The α-values of samples G50–G80 (with CaO and Al_2_O_3_ containing phases) were lower than those of samples G10–G40, free from Al_2_O_3_ containing phases and composed of crystalline phases with high CTE values, which are β-wollastonite, para-wollastonite and diopside.

It is worth mentioning that β-wollastonite (CaSiO_3_) contains triclinic wollastonite and monoclinic parawollastonite. These forms are not easily differentiated except for single-crystal X-ray investigation [46]. The stabilities of β- and para-wollastonite phases are likely to be very similar; because of the presence and intergrowth of both forms [47].

## 4. Conclusions

1. The high-performance glass–ceramic materials were successfully obtained through a wollastonite–cordierite system based on industrial waste and natural raw materials.
2. The present results showed that the by-pass cement dust could be used in quantities that may exceed 60% of the batch weight to produce glass–ceramic materials. This application may pave the way for the disposal of this waste in an environmentally friendly manner. By-pass cement and natural raw materials can be used successfully in preparing glass–ceramic materials without any chemical additives.
3. These glass–ceramic materials have been used for different purposes, such as floor and wall tiles, benchtops, sewer pipes, and many others.
4. Cleaning, environmental protection, and public health preservation by getting rid of by-pass cement dust through the preparation of glass–ceramic materials.
5. It was found that β-wollastonitte is formed at low temperatures, and this helps to save energy and then turns into parawollastonite at higher temperatures. Accordingly, glass–ceramic materials containing β-wollastonite have a better economic value due to their formation at low temperatures.
6. A fine-grained microstructure is obtained in the samples that are cordierite-rich.
7. Increasing the time of heat-treatment at endothermic temperature helped form fine-grained microstructures.
8. Obtaining glass–ceramic materials with low thermal expansion ranging from 6.216 to 2.618 × 10^−6^ (in the range of 20–700 ° C) decreased with an increasing percentage of cordierite.

## Figures and Tables

**Table 1:** Chemical analyses of raw materials used in the batch preparation.

Oxide wt %	Magnesite (Al-Nasr Mining Co.)	Silica Sand Abu-Zenima (Sinai)	Kaolin El-Tih (Sinai)	By-Pass Cement
SiO_2_	0.54	99.20	44.20	6.12
Al_2_O_3_	1.02	0.28	37.75	2.58
Fe_2_O_3_	0.48	0.03	0.93	3.37
TiO_2_	trace	trace	1.85	0.21
CaO	6.31	0.10	0.82	55.96
MgO	40.35	trace	0.52	0.84
Na_2_O	trace	trace	1.15	0.29
K_2_O	trace	trace	0.72	0.73
LOI at 1000 °C	50.98	0.40	13.01	25.11

**Table 2:** Chemical compositions and the corresponding batches (wt%) of the investigated glasses.

Glas No.	Nominal Phase * Composition (wt%)	Oxide wt%				Batch Ingredients (wt%)			
		SiO_2_	Al_2_O_3_	CaO	MgO	By-Pass	Mag.	Kaolin	Sand
G10	10%cord. + 90% wol.	51.68	3.49	43.45	1.38	60.2	1.4	3.21	35.19
G20	20%cord. + 80% wol.	51.65	6.97	38.62	2.76	53.19	4.15	11.11	31.54
G30	30%cord. + 70% wol.	51.61	10.46	33.8	4.13	46.29	6.87	18.95	27.9
G40	40%cord. + 60% wol.	51.57	13.95	28.97	5.51	39.43	9.57	26.68	24.31
G50	50%cord. + 50% wol.	51.54	17.43	24.14	6.89	32.63	12.22	34.28	20.87
G60	60%cord. + 40% wol.	51.54	20.92	19.31	8.27	25.98	14.87	41.89	17.26
G70	70%cord. + 30% wol.	51.47	24.4	14.48	9.65	19.38	17.47	49.34	13.8
G80	80%cord. + 20% wol.	51.43	27.89	9.66	11.02	12.84	20.29	56.35	10.53

Where, * cord = cordierite, wol. = wollastonite, Mag. = magnesite.

**Table 3:** Thermal expansion coefficient and phases developed of the investigated samples.

Glass No.	Linear Expansion Coefficient (ἀ) × 10^−6^/°C			Phases Developed
	50–300 °C	50–500 °C	50–700 °C	
G10	4.0838	5.9616	6.2161	Β-woll.
G20	2.7820	3.4909	5.2155	Β-woll.
G30	2.0008	3.0399	4.5930	Diop. and parawoll.
G40	1.9857	3.0385	4.5721	Parawo. and Diop
G50	1.3134	3.0764	4.5671	An. and Diop
G60	1.2896	2.7932	3.6538	An. and Diop
G70	1.2126	2.4522	3.3364	An. and Diop
G80	0.1478	2.2153	2.6181	Cord. and An.

Where. Β-woll. = Β-wollastonite, Diop. = Diopside, parawoll. = Parawollastonite, An. = Anorthite, Cord. = Cordierite.

## Data Availability

The data of this work is available for first author after puplication.
